# No Exchange of Picornaviruses in Vietnam between Humans and Animals in a High-Risk Cohort with Close Contact despite High Prevalence and Diversity

**DOI:** 10.3390/v13091709

**Published:** 2021-08-27

**Authors:** Lu Lu, Jordan Ashworth, Dung Nguyen, Kejin Li, Donald B. Smith, Mark Woolhouse, on behalf of the VIZIONS Consortium

**Affiliations:** 1 Usher Institute, University of Edinburgh, Edinburgh EH9 3FL, UK; jordan.ashworth@ed.ac.uk (J.A.); mark.woolhouse@ed.ac.uk (M.W.); 2 Nuffield Department of Medicine, University of Oxford, Oxford OX3 7BN, UK; dung.nguyen@ndm.ox.ac.uk (D.N.); donald.smith@ndm.ox.ac.uk (D.B.S.); 3 Institute of Evolutionary Biology, University of Edinburgh, Edinburgh EH9 3FL, UK; likejin2019@gmail.com

**Keywords:** picornavirus, animal–human interface, metagenomic sequencing, rats, bats

## Abstract

Hospital-based and community-based ‘high-risk cohort’ studies investigating humans at risk of zoonotic infection due to occupational or residential exposure to animals were conducted in Vietnam, with diverse viruses identified from faecal samples collected from humans, domestic and wild animals. In this study, we focus on the positive-sense RNA virus family *Picornaviridae*, investigating the prevalence, diversity, and potential for cross-species transmission. Through metagenomic sequencing, we found picornavirus contigs in 23% of samples, belonging to 15 picornavirus genera. Prevalence was highest in bats (67%) while diversity was highest in rats (nine genera). In addition, 22% of the contigs were derived from novel viruses: Twelve phylogenetically distinct clusters were observed in rats of which seven belong to novel species or types in the genera *Hunnivirus, Parechovirus, Cardiovirus, Mosavirus* and *Mupivirus*; four distinct clusters were found in bats, belonging to one novel parechovirus species and one related to an unclassified picornavirus. There was no evidence for zoonotic transmission in our data. Our study provides an improved knowledge of the diversity and prevalence of picornaviruses, including a variety of novel picornaviruses in rats and bats. We highlight the importance of monitoring the human–animal interface for possible spill-over events.

## 1. Introduction

Viruses of zoonotic origin are a significant public health concern worldwide having given rise to a number of high-profile health emergencies. Examples of diseases which are known or presumed to have been transmitted from animals to humans include avian influenza A virus, MERS and SARS-CoV-2 [1,2]. Direct or indirect contact between humans and animals is essential for a successful cross-species transmission [3]. The incidence of zoonotic disease is likely to be higher in regions where there is frequent contact between humans and animals.

The multidisciplinary surveillance program Vietnam Initiative on Zoonotic InfectIONS (VIZIONS) project was conducted to explore the zoonotic potential of viruses across different rural sites in Vietnam [4]. Studies focussed on small-scale farmers who house several different animal species within a small area with minimum biosecurity and may also eat wild animals or be involved in commercial wildlife farming, processing, or selling. Such activities increase the likelihood of pathogen transfer between wildlife, humans, and domestic animals. Another component of VIZIONS was hospital-based surveillance. Metagenomic sequencing was used to identify viruses in samples collected from 2012 to 2016 from humans who presented with enteric disease and potential zoonotic contacts, these being both domestic and wild animals. Subsequent phylogenetic analysis was used to investigate the viruses identified in these samples, including their spatial and temporal spread through human and animal populations and the inference of any zoonotic transmission events.

Picornaviruses are non-enveloped viruses with a positive-sense single-stranded RNA genome of 6.7–10.1 kb containing a single long open reading frame (ORF) [5]. Picornaviruses are globally distributed and infect vertebrates of all classes [6]. Sixty-eight genera and 158 species are currently recognised by the ICTV [5]. Of these, members of nine genera and 24 species are known to be human-infective [7]. Previous VIZIONS studies have reported a variety of novel picornaviruses in both humans and animals, including diverse members of the species *Enterovirus G* types in domestic pigs and farmed wild boar [8], and of the genus *Kobuvirus* in rodents and bats [9]. However, there may still be novel and unknown picornaviruses present in animals, whose existence may increase the scope for further transmission events between humans and animals, where there is close contact. These transmission events have the potential to become the origin of emerging infectious diseases. The aims of this VIZIONS study were to describe the prevalence and genetic diversity of picornaviruses present in wide range of hosts at the animal–human interface; to identify novel picornaviruses circulating in animals and humans and to explore possible animal sources of picornavirus infections in humans.

## 2. Materials and Methods

### 2.1. Samples

For the study, 2100 faecal samples collected from human and non-humans in Vietnam between year 2012 and 2016 were subjected to a viral diagnostic algorithm and put through a next generation sequencing (NGS) process [4,10]. Of these, 1258 samples were obtained from human subjects: 707 samples were collected from patients admitted to hospital with diarrhoea and 551 samples were obtained from a high-risk cohort in Dong Thap province (farm households, animal health workers, abattoir workers, poultry slaughterers, rat traders). In parallel, 842 samples were obtained from a variety of non-human animals in Dong Thap including: rats which were farmed or traded in the live markets (*n* = 315) and 93% were from rat species *Rattus argentiventer* (commonly known as ricefield rat and is a species of rat found throughout Southeast Asia); bats whose faeces were collected from roosting sites for trading (*n* = 179); domestic pigs (*n* = 270) and farmed wild boar (*n* = 15). A smaller number of samples were collected from domestic dogs (*n* = 45), cats (*n* = 13), monkeys (*n* = 3) and goats (*n* = 2). Information on host species, sampling date, location and other attributes is available for all samples [11].

### 2.2. Metagenomic Analysis

Reads from 2100 metagenomics samples had adapters and low-quality bases removed using Cutadapt [12]. Reads which mapped, when queried against a custom database consisting of human, pig, rat and several hundred representative bacterial genomes using KRAKEN, were removed [13]. Remaining reads were assembled into contigs using MetaSPAdes [14]. Each of the 2100 samples were assembled individually using the default parameters. Contigs <700 nucleotides were removed. Remaining contigs were queried against a custom viral protein database containing all protein sequences available on GenBank as of 5 May 2021 that had the *Picornaviridae* taxonomic ID (NCBI: txid12058) using the DIAMOND BLASTX ultra-sensitive algorithm [15]. An e-value cut-off of <=10 × 10^−50^ and an alignment cut-off of >=500 amino acids were applied for hits to be categorised as positive. Contigs with positive hits were annotated using Geneious Prime 2021.1.1 (https://www.geneious.com, accessed on 2 April 2021).

### 2.3. Genomic and Phylogenetic Analysis

Nucleotide sequences of genomes and amino acid sequences of open reading frames (ORFs) were aligned with the reference sequences downloaded from ICTV and NCBI database using MAFFT [16]. The best substitution model was evaluated using the Model Selection package. Maximum-likelihood trees were generated with 1000 bootstrap replicates with IQTREE [17]. Time-scaled phylogenies for the VP1 gene were generated using the Bayesian Monte Carlo Markov Chain (MCMC) method implemented in BEAST v.1.10 [18]. The combinations of substitution models, clock models, and population size models were evaluated by stepping-stone methods: a SRD06 nucleotide substitution with uncorrelated lognormal relaxed molecular clocks and a constant-population coalescent process prior. Priors of substitution rate were chosen according to published evolution rates from related genera in the family *Picornaviridae* [8], using the mean rate of 2 × 10^−^^3^^ ^ sub/site/year with SD = 1 × 10^−^^3^. MCMC chains were run for 100 million steps, sampled every 10,000 states with 10% burn-in. MCMC convergence and effective sample size of parameter estimates were evaluated using Tracer 1.7 (http://beast.bio.ed.ac.uk, accessed on: 24 May 2021). Maximum clade credibility (MCC) trees were summarized using Tree Annotator and visualized using FigTree v1.4.4 (http://tree.bio.ed.ac.uk/software/figtree/, accessed on: 1 June 2021). The overall rates of evolutionary change (ns/s/y, nucleotide substitutions per site per year) and tree root ages were estimated simultaneously. The pairwise amino acid (aa) and nucleotide (nt) identities were calculated using Geneious Prime 2020.1.1.1 (https://www.geneious.com, accessed on 15 June 2021). The genetic scan of complete polyprotein coding sequences was using simplot in R (v.3.6.1). Metadata of picornavirus sequences found in this study and sequences from other study used in phylogenetic trees (Figures 5–7) are listed in Supplementary Table S1.

## 3. Results

### 3.1. High Prevalence of Diverse Picornaviruses

Virus contigs were obtained from 2100 faecal samples following the processing of raw metagenomic data and analysis of the de novo assembled sequences. BLASTX analysis of these contigs against the NCBI protein database and a further data clean step (see Methods) revealed 2370 contigs (lengths 701–8869 nt) that were closely related to members of the family *Picornaviridae*, these being derived from 23% (*n* = 493) of faecal samples.

**Figure 1:**
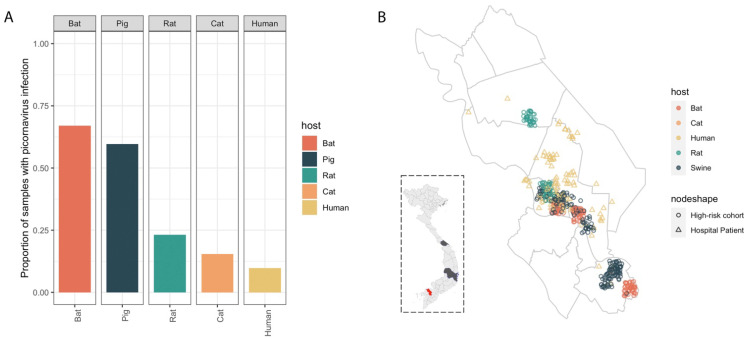
Prevalence of picornavirus contigs by host. (**A**) Proportion of samples with picornavirus sequences detected in each host type. (**B**) Samples with picornavirus sequences identified in Dong Thap province (highlighted in red in dashed square). Symbol colours indicate host (used consistently in all figures), and for human samples symbol shape indicates those from the risk cohort study (circles) and hospital patients (triangles).

Picornavirus sequences were detected most frequently in samples from bats (67%) and pigs (60%), but were also detected in rats (23%), cats (15%) and humans (10%) (Figure 1A). No picornavirus was detected from samples of monkeys, dogs, or goats, which may be due to low number of samples for these hosts. Within Dong Thap, the geographic locations of human and non-human samples with picornavirus identified overlapped in the central area (Figure 1B).

**Figure 2:**
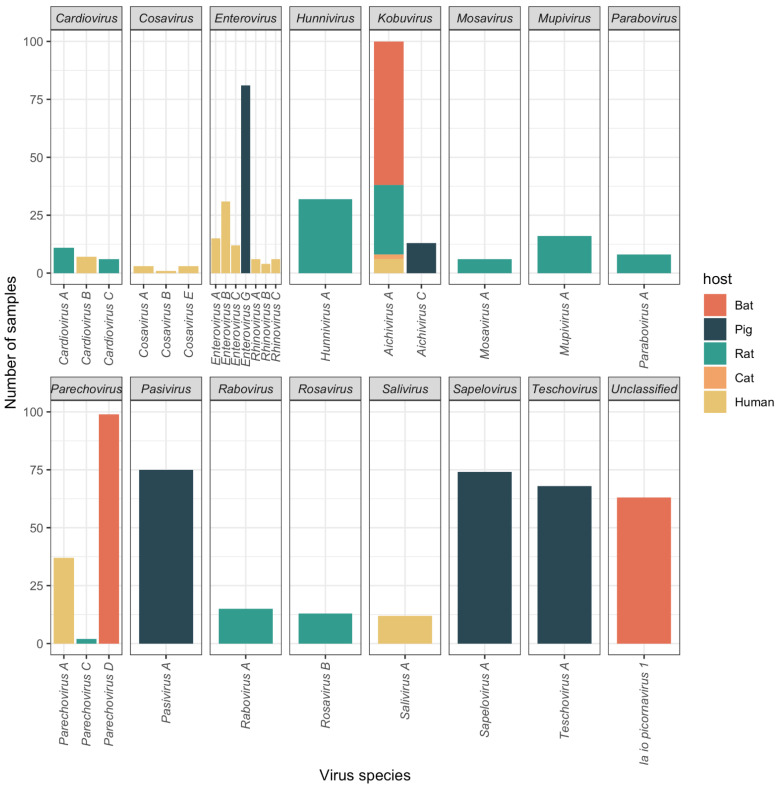
Prevalence of picornavirus species in different hosts by DIAMOND BLASTX top hit.

Using the current demarcation criteria for genera and species within the family *Picornaviridae*, the 2370 contigs belong to 15 genera (29 species) and one groups with an unclassified picornavirus (Figure 2). In our study, the only picornavirus species that was detected in more than one host was *Aichivirus A* (belonging to genus *Kobuvirus*). Members of *Aichivirus A* are known to infect a wide range of hosts, with human aichivirus as a distinct type (AiV-A1) from the other types found in nonhuman animals [9].

Contigs assigned to different picornavirus genera and species were unequally distributed among hosts (Figure 2). Members of the genera *Hunnivirus*, *Mosavirus*, *Mupivirus*, *Parabovirus*, *Rabovirus* and *Rosavirus* were only detected in rats, members of the genera *Pasivirus*, *Sapelovirus* and *Teschovirus* were only detected in pigs, members of the genera *Cosavirus* and *Salivirus* were only detected in humans, and an unclassified picornavirus related to Ia io picornavirus 1 [5] was only detected in bats. Similarly, members of the genus *Cardiovirus* were only found in rats and humans, members of the genus *Enterovirus* in pigs and humans, and members of the genus *Parechovirus* in rats, bats, and humans. *Kobuvirus* was the only genus for which viruses were detected in rats, bats, pigs, cats, and humans.

Within each host, the relative abundance of viruses in different taxa varied considerably. For example, for humans, members of the species *Parechovirus A* and *Enterovirus B* were detected in >20% of samples, with >5% of samples containing members of the species *Enterovirus A*, *Enterovirus C* or *Salivirus A.* Similarly, members of 10 different species were detected in >5% of rat samples, three different species in >28% of bat samples and six different species in >5% of pig samples. Detection of members of more than one picornavirus species from the same sample was most common in bats (57%), pigs (55%) and rats (42%), but less frequent in humans (15%).

### 3.2. Putative Novel Picornaviruses

The current demarcation criteria between picornavirus genera are genetic identities of <34% amino acid (aa) for the polyprotein and P1 proteins, and <36% for the 2C and 3CD proteins, while between species the criteria are <70% aa identity for the polyprotein and P1, and <80% identity for 2C and 3CD [5,19]. Criteria for distinguishing types within a picornavirus species are less well established, as they are usually based on phylogenetic analysis; for enteroviruses and rhinoviruses, <75% and <87% nt identity in VP1 gene, respectively [20,21,22]. Accordingly, we have adopted a threshold of <75% nt identity in VP1 gene in combination with phylogenetic analysis to identify novel types.

**Figure 3:**
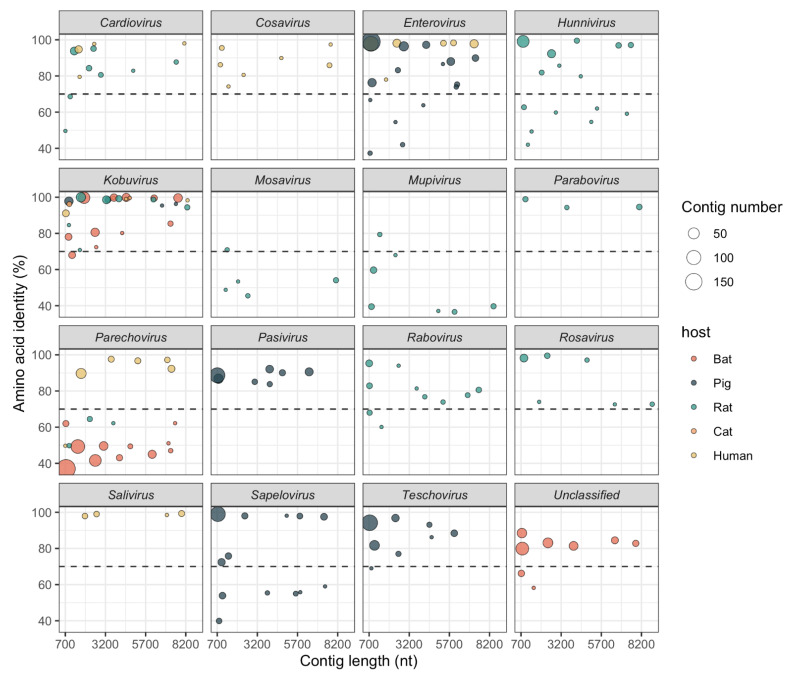
Identity of contigs to the closest related sequence in NCBI. Circle size indicates the number of contigs, and fill colour indicates the host. Contigs with <70% aa identity to reference sequences (dotted line) may represent members of novel species.

Amino acid (aa) identities between the 2370 picornavirus contigs (lengths 701–8869 nt) and the closest picornavirus reference genomes were <70% for 523 of them (22%), mostly from bats (*n* = 428) and rats (*n* = 52). Although these contigs do not always correspond to regions used to demarcate taxa and types as described above, they may represent novel picornavirus species in the genera *Cardiovirus*, *Parechovirus*, *Enterovirus, Hunnivirus*, *Mupivirus*, *Kobuvirus*, *Mosavirus*, *Sapelovirus*, *Rabovirus*, *Teschovirus*; other sequences are distantly related to an unclassified picornavirus (Figure 3).

### 3.3. Evolutionary Characteristics of Putatively Novel Picornaviruses Found in Rats and Bats

**Figure 4:**
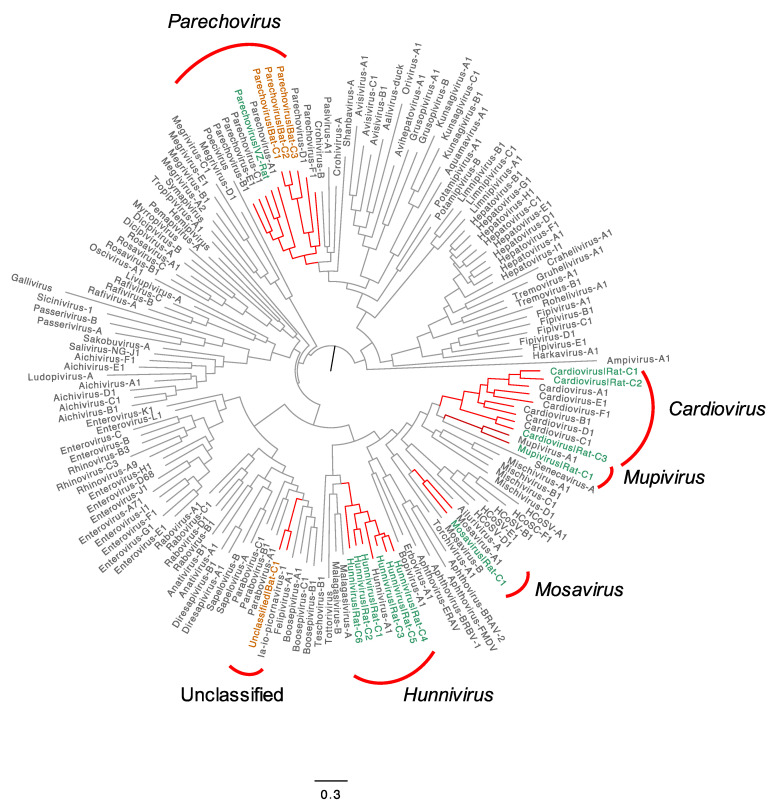
Phylogenetic analyses of putatively novel species or types found in rats and bats and representative members of the family *Picornaviridae* based on the coding region of P1. The branches of 5 genera and an unclassified picornavirus with novel clusters (labelled with C)/sequences from rats (green in tips) and bats (orange in tips) found in Vietnam are highlighted in red. The trees are constructed using maximum likelihood methods with HKY + G4 + I model and 1000 bootstraps. Scale bar indicates number of nucleotide substitutions per site.

We conducted a more detailed analysis of sequences belonging to five selected genera (*Cardiovirus*, *Parechovirus*, *Hunnivirus*, *Mupivirus*, *Mosavirus*) and sequences similar to an unclassified picornavirus, which are likely to represent novel species or types in rats and bats (Figure 3). Phylogenetic trees were generated to determine their relationship to known picornaviruses (Figure 4).

Overall, we found 12 phylogenetically distinct clusters in rats, seven of which are members of putative novel species/types in the genera *Hunnivirus*, *Parechovirus*, *Cardiovirus*, *Mosavirus* or *Mupivirus*; four distinct clusters found in bats belong to one novel species in the genus *Parechovirus*, and one cluster is related to an unclassified picornavirus. The detailed genetic and phylogenetic analyses of individual genus are described in Section 3.3.1, Section 3.3.2, Section 3.3.3, Section 3.3.4, Section 3.3.5 and Section 3.3.6.

#### 3.3.1. Diversity of Hunniviruses in Rats

The genus *Hunnivirus* has a single species, *Hunnivirus A,* members of which belong to nine types (HuA-A1 to A9) detected in rats (HuV-A4 to A9), cattle (HuV-A1) and sheep (HuV-A2 and A3). An additional isolate from cat is unclassified [5].

**Figure 5:**
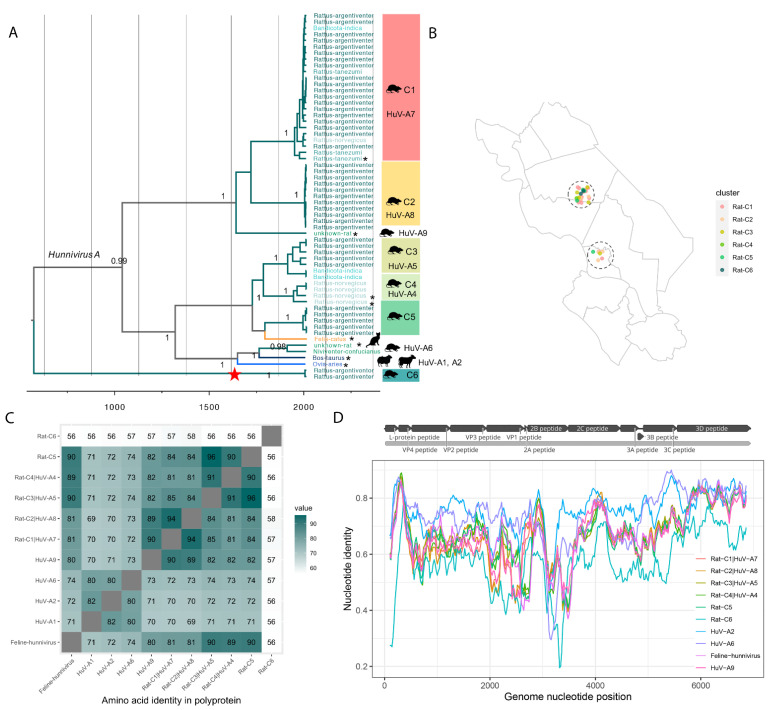
*Hunnivirus* sequences in rats and other hosts. (**A**) Simplified MCC tree representing the time-scale phylogeny of hunniviruses. Bayesian time-scaled tree of VP1 sequences detected in Vietnamese rats and reference sequences in NCBI (highlighted in asterisk). Different host species are labelled on the tips and distinct by colours and symbols (rat in different shades of green, cat in orange, bovine and ovine in dark blue); the six clusters found in Vietnamese rats (Rat-C1-C6) are labelled on the right; the internal node of Rat-C6, a putative member of a novel species, is highlighted with red star. (**B**) Locations of rat samples with hunniviruses, coloured by different clusters identified in the phylogenetic tree. (**C**) Amino acid identities of polyprotein among rat clusters and other hosts. (**D**) Nucleotide distance scan across the polyprotein coding region of rat clusters (Rat-C1 to C6) against HuA-A1 and comparing to other known hunnivirus types.

A time-scaled phylogenetic tree was produced using full length VP1 genes from representatives of each virus type (*n* = 9), Vietnamese sequences identified in VIZIONS studies (*n* = 50) and sequences from other hosts/countries retrieved from NCBI (*n* = 9) (Figure 5A).

Hunnivirus sequences from Vietnamese rats (*Rattus argentiventer, Bandicota indica, Rattus tanezumi* and *Rattus norvegicus*, all purchased from live markets in two districts of Dong Thap province) grouped into six clusters (Hunnvirus|Rat-C1 to Hunnvirus|Rat-C6) with estimated origin time (TMRCA) between 12 to 125 years ago and no geographic separation between different clusters (Figure 5A,B), indicating that diverse hunniviruses co-circulate and have been wide spread in rat populations in Dong Thap for a long time. Among the six clusters, four of them include (>81% identity within a type) known types previously described only or mainly from Vietnam: Rat-C1 (*Rattus argentiventer, Bandicota indica, Rattus tanezumi and Rattus norvegicus*; also (MF352430 (from *Rattus tanezumi* in China) belongs to HuV-A7, Rat-C2 (*Rattus argentiventer*) belongs to HuV-A8, Rat-C3 (*Rattus norvegicus* and *Bandicota indica*) belongs to HuV-A5 and Rat-C4 belongs to HuV-A4 (*Rattus norvegicus;* also from the same species in USA (KJ950971) and China (MW417242)).

Sequences belonging to Rat-C5 (>93% nt identity within the group, 73–74% with other hunniviruses) were isolated from *Rattus argentiventer*; their nearest relative is an unclassified hunnivirus sequence (MF953886) isolated from a cat [5] (Figure 5A). Two genomes isolated from *Rattus argentiventer* in the same market grouped into a further cluster (Rat-C6). Sequences in this cluster share 44–51% identity in VP1 nt sequence and 56–58% identity in polyprotein aa sequence (Figure 5C), suggesting that Rat-C6 may represent a novel hunnivirus species. A genetic distance scan (Figure 5D) across the coding region demonstrated identities between the representative Rat-C6 genome and hunnivirus genomes in other rat clusters and other hosts. In the P1 region, the sequence identity of Rat-C6 compared to other rat clusters Rat C1-6 (56–59%), which is similar to that for hunniviruses in cat (56%), cattle (55%) and sheep (55%). The 3D (RdRp) region of Rat-C6 has 76–78% aa identity with the reference sequences from other rat clusters as well as other hosts.

#### 3.3.2. Diversity of Parechoviruses in Bats and Rats

The genus *Parechovirus* includes six species (*Parechovirus A*, *Parechovirus B*, *Parechovirus C*, *Parechovirus D*, *Parechovirus E* and *Parechovirus F*), within which 29 types are distinguished, with humans (*Parechovirus A*) and rodents (*Parechovirus B* and *Parechovirus C*) as the major hosts. Parechoviruses have also been identified from ferrets (*Parechovirus D*), bats (*Parechovirus D*), birds (*Parechovirus E*) and lizards (*Parechovirus F*).

**Figure 6:**
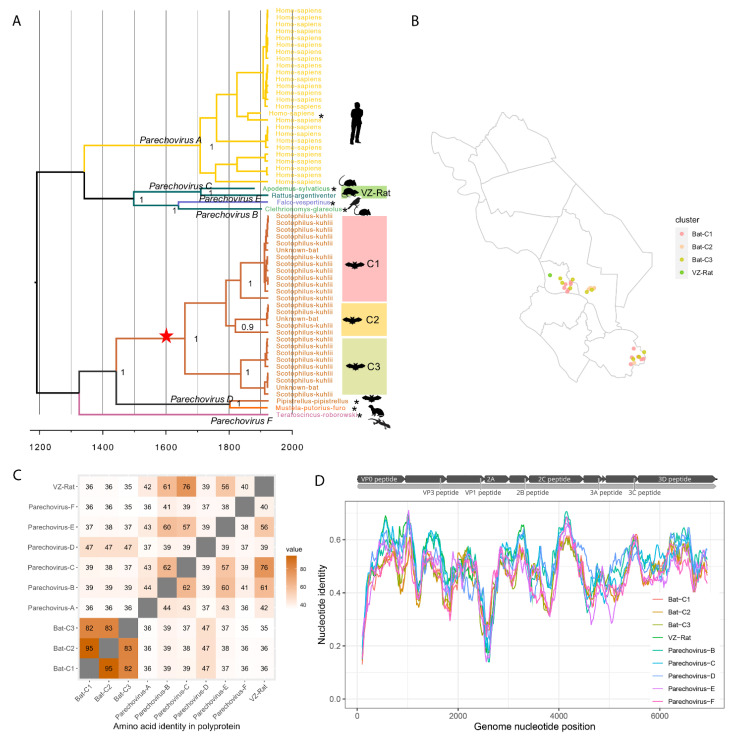
*Parechovirus* sequences in bats, rats, and other hosts. (**A**) Simplified MCC tree representing the time-scale phylogeny of parechoviruses. Bayesian time-scaled tree of VP1 sequences detected in Vietnamese rats and reference sequences in NCBI (highlighted in asterisk). Currently identified species in the genus *Parechovirus* are labelled on the nodes. Different host species are indicated on the tips and distinct by colours and symbols; sequences from a Vietnamese rat (Parechovirus|VZ-Rat) and the clusters (Parechovirus|Bat-C1 to C3) found in Vietnamese bats are labelled on the right; the internal node of the putatively novel species found in bats is highlighted with red star. (**B**) Locations of bat and rat samples with parechovirus sequences, coloured by different clusters identified in the phylogenetic tree. (**C**) Genetic identities in polyprotein among bat and rat sequences found in Vietnam comparing to reference sequences of species in the genus *Parechovirus*. (**D**) Genetic distance scan across the entire polyprotein coding region of bat clusters and rat sequence found in Vietnam, comparing to reference sequences of *Parechovirus A*.

A time-scaled tree of parechovirus VP1 gene including 53 sequences obtained in this study and the reference sequences of each species (*n* = 7) revealed that the 25 sequences from humans, all from hospitalized children (0–3 years old) with diarrhoea belong to *Parechovirus A* (Figure 6A). However, the sequences sampled from bats (mainly *Scotophilus kuhlii* from bat roosts in three regions in Dong Thap) separate into three adjacent clusters (Parechovirus|Bat-C1 to Parechovirus|Bat-C3) with a TMRCA of 294 years ago (95% HPD 102 to 536 years), with VP1 nucleotide identities of (71–74% (C1/C2), 59–65% (C1/C3), and 60–65% (C1/C3), suggesting that they are members of three different types of a novel species (Figure 6A,B). The closest relation to these Vietnamese bat sequences is QAPp32 (lMK348056) (48–59%, 60–62% and 57–58% aa identity in the polyprotein, P1 and 3D regions, respectively), which was recently sampled from a bat *(Pipistrellus pipistrellus*) in China and belongs to *Parechovirus D* [23].

In addition, we found one novel sequence (Parechovirus|VZ-Rat, 22057×67-9) from a rat (*Rattus argentiventer*) which groups with Sebokele-virus-1, a member of the species *Parechovirus C*, and isolated from wood mice (*Apodemus sylvaticus*) in Africa [24], with which it shared 62% nucleotide identity (Figure 6A). The partial polyprotein of the rat virus (length = 3670 nt, with complete P1 and partial P2 coding regions) shared 78% aa identity to Sebokele-virus-1, and 75% identity in the P1 region (Figure 6C,D).

#### 3.3.3. Diversity of Cardioviruses in Bats and Rats

The genus *Cardiovirus* includes the species *Cardiovirus A, Cardiovirus B, Cardiovirus C, Cardiovirus D, Cardiovirus E* and *Cardiovirus F*, members of which belong to 32 types, with rodents as the natural reservoirs.

**Figure 7:**
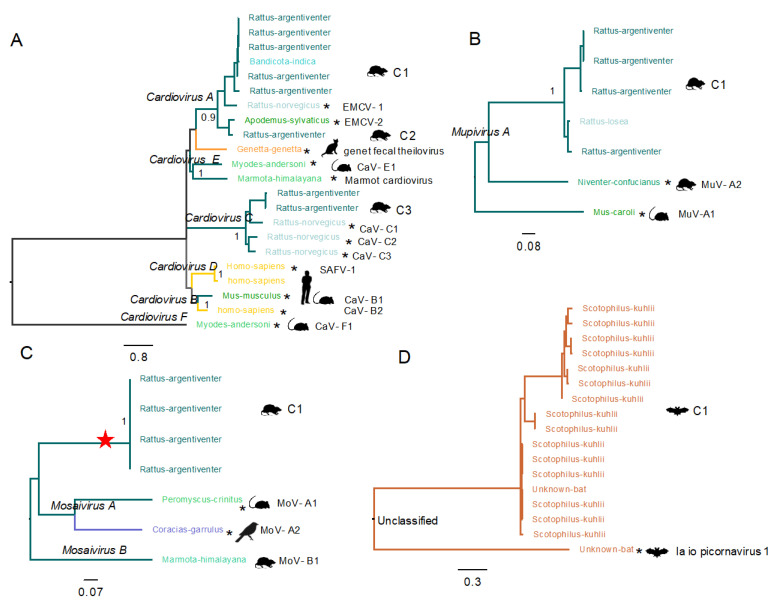
Phylogenetic relationships between picornaviruses found in rats, bats, and other hosts. (**A**) *Cardiovirus,* (**B**) *Mupivirus,* (**C**) *Mosavirus* and (**D**) unclassified picornavirus. Maximum likelihood tree of VP1 sequences detected in Vietnam and reference sequences in NCBI (highlighted with an asterisk). Virus species are labelled on the nodes. Different host species are indicated on the tips and distinguished by colours and symbols; sequences from the clusters found in Vietnamese are labelled on the right. The internal node of members of a putative novel mosavirus species is highlighted with red star.

In the VP1 gene tree, a sequence isolated from an enteric patient (1 year old) grouped with the reference genome for the species *Cardiovirus D* (Saffold virus 1, EF165067) (Figure 7A), also displaying a 98% aa identity in polyprotein aa sequence.

Cardiovirus sequences from three rodent species (*Rattus argentiventer, Rattus norvegicus* and *Bandicota indica*) sampled at in the same market in Dong Thap fall into three clusters (Cardiovirus|Rat-C1 to Cardiovirus|Rat-C3) (Figure 7A). Rat-C1 comprises six sequences (five from *Rattus argentiventer* and one from *Bandicota indica*) that belong to the species *Cardiovirus A*, sharing 64–66% identity in the VP1 nucleotide sequence and 73% in the polyprotein aa sequence to the closest related reference sequence cardiovirus A1 (EMCV-1, isolated from *Rattus norvegicus* in 1992) (Figure 7A). The cluster Cardiovirus|Rat-C2 contains a partial genome (22084×1-3992) also belonging to Cardiovirus A, sharing 79% identity in the VP1 nucleotide sequence and 82% in the polyprotein aa sequence with the reference sequence cardiovirus A2 (EMCV-2, isolated from *Apodemus sylvaticus* in 2005). Interestingly, Cardiovirus|Rat-C2 was isolated from a rat sample together with a sequence (22084×1-3987) in Cardiovirus|Rat-C1, indicating that multiple cardiovirus types can infect one individual.

The cluster Cardiovirus|Rat-C3 comprises two sequences isolated from *Rattus argentiventer* from the same date and place (Figure 7A) and group (sharing 65% identity in VP1 gene nucleotide sequence and 79% identity in the polyprotein aa sequence) with viruses isolated from *Rattus norvegicus* in the US [25].

The results suggest that Cardiovirus|Rat-C1 and Cardiovirus|Rat-C3 may represent new types in the species *Cardiovirus A* and *Cardiovirus C*, respectively.

#### 3.3.4. A New Type of Mupivirus in Rats

Memebrs of the genus *Mupivirus* are adjacent to those in the genus *Cardiovirus* in the phylogeny of P1 coding region (Figure 4). *Mupivirus* is a newly designated genus in the family *Picornaviridae* represented by a single species (*Mupivirus A*). Two types are distinguished: mupivirus A1 (RtRn-PicoV/YN2014 (KY432924.1)) and mupivirus A2 (RtNn-PicoV/HuB2015-2 (KY432934.1)), isolated in China from Ryuku mice (*Mus caroli*) and Chinese white-bellied rats (*Niventer confucianus*), respectively [26].

Divergent mupivirus genomes were isolated from two different rat species (four from *Rattus argentiventer* and one from *Rattus losea*) at two separate locations in Dong Thap. These viruses encode a Leader protein that is 93 aa longer than that of other viruses in the genus. A VP1 gene tree shows that these mupiviruses grouped into a distinct cluster (Mupivirus|Rat-C1, 87–99% VP1 nt identity within the group), sharing 56–57% nucleotide identity with that of mupivirus A1 and 60–61% identity with that of mupivirus A2.

The Mupivirus|Rat-C1 polyprotein shares ~70% aa identity with mupivirus A1 and A2, 74–78% aa identity in the P1 region and 84–88% aa identity in the 3D region (Figure 7B). This suggests that the viruses in Mupivirus|Rat-C1 may belong to a new type of *Mupivirus A*.

#### 3.3.5. Novel Species of Mosavirus in Rats

The genus *Mosavirus* includes two species (*Mosavirus A* and *Mosavirus B*), whose members fall into three types: mosavirus A1 and mosavirus A2 were detected, respectively, in faeces of the canyon mouse (*Peromyscus crinitus*) [27] and the European roller (*Coriacas garrulus*) [28] while mosavirus B1 was identified in the intestinal contents of captured Himalayan marmots (*Marmota himalayana*) in China [29].

A VP1 tree reveals a cluster (Mosavirus|Rat-C1, >99% identity in VP1 nt sequence) of four mosavirus genomes derived on the same date and place in Dong Thap from faeces of *Rattus argentiventer* (Figure 7C). These viruses differed from reference aa sequences of viruses in the species *Mosavirus A* and *Mosavirus B* by 47–58% in the polyprotein, 45–59% in the P1 region and 53–70% in the 3D region, suggesting that they represent a novel species in the genus *Mosavirus*.

#### 3.3.6. Unclassified Picornavirus in Bats

Ia io picornavirus 1 (JQ814852.1) is an unclassified picornavirus identified in China in 2010 during a study of the bat virome [30]. A phylogenetic tree based on the P1 coding region of the reference sequences of members of the family *Picornaviridae* shows that Ia io picornavirus 1 is adjacent to members of the genus *Felipivirus* (isolated from cats) with 55% aa identity to felipivirus A1 (JN572115) (Figure 4).

A cluster of sequences (Unclassified|Bat-C1) isolated from bat faeces collected from three bat roosts in Dong Thap are 82–93% identical to each other in VP1 gene nt sequence, but only 71–72% identical to Ia io picornavirus 1 (Figure 7D). Similarly, genomes in the cluster Unclassified|Bat-C1 share 82–83% identity to Ia io picornavirus 1 in the polyprotein aa sequence, 72–73% identity in the P1 region aa sequence, and 87–88% identity in 3D region aa sequence. These results suggest that Unclassified|Bat-C1 represents a new type related to Ia io picornavirus 1.

## 4. Discussion

The VIZIONS project identified and assessed the diversity of viruses in 2100 samples from humans and a range of animals with close human contact in a high-risk region for zoonotic disease.

Bats and rats are the reservoir of many zoonotic viruses, and a range of human-infective viruses can be transmitted directly to humans or through an intermediate host, examples including hantaviruses and coronaviruses. Trade in wildlife might play a role in the origin of COVID-19, and viruses closely related to SARS-CoV-2 have been identified in bats and pangolins, both traded widely. A recent study showed rodents in Vietnam had evidence of murine coronavirus infection, with prevalence increasing along a wildlife trading route and being highest at markets [31].

In our study, picornaviruses were most commonly found in bats (67%) and were most diverse in rats (members of nine genera). More than one fifth of the picornavirus contigs belong to novel picornavirus species (or types) in five different genera; these sequences mainly deriving from bats and rats.

Two virus discovery studies on wild rodents were conducted in multiple regions in China during the same time frame as VIZIONS: one study found that 2% of wild rodent samples contained picornavirus sequences, with these viruses belonging to seven picornavirus genera [26]. Another study found seven novel sequences from six rodent species including one potentially representing a novel genus closely related to *Hepatovirus,* as well as new species in the genera *Rosavirus, Hunnivirus*, and *Enterovirus* [32].

Another study identified viruses from five picornavirus genera in 133 urban rats (*Rattus norvegicus*) in New York City, with members of the genus *Kobuvirus* (66%) detected most frequently [25]. These authors concluded that picornavirus diversity in the virome of rats is greater than for any other non-human mammal species, suggesting that rats may be a significant reservoir for picornaviruses, at least in the urban environment. Our findings are consistent with this conclusion in that rats were infected with more diverse picornaviruses (members of nine picornavirus genera) than any of the other hosts sampled.

Although the first hunnivirus was isolated in 1969 [33], it was only recently placed in its own genus as a member of the only species. A limited number of hunnivirus genomes have been identified from rodents in the USA and China [25,26,32]. In comparison, we identified six distinct clusters of hunniviruses in 22% of rats of four different species purchased from wildlife markets in Dong Thap, Vietnam. Four of these viruses belong to four known types of clusters mainly composed of sequences retrieved from Vietnam as part of the VIZIONS study. One cluster of sequences includes a hunnivirus distinct from all known hunniviruses, and another cluster is closely related to a recently identified hunnivirus from a cat with diarrhoea in China [34]. Our findings indicated that rats might act as the major reservoir of diversified hunnviruses.

Members of the species *Cardiovirus A* and *Cardiovirus B* have been detected in human samples [35]. Viruses belonging to *Cardiovirus C* and *Cardiovirus B* (with a prevalence of 28% and 14%, respectively) were detected from urban *Rattus norvegicus* in the US [25]. In comparison, the overall detection frequency of cardioviruses in two rat species (*Rattus argentiventer* and *Bandicota indica*) in this study was 12%, and the viruses we identified were putatively new types of *Cardiovirus A* and *Cardiovirus C*. No viruses of the species *Cardiovirus B* were found in either rats or humans in our study. Similarly, members of the species *Parechovirus B* (Ljungan virus) have been reported in rodents such as voles and mice [26,36,37,38,39,40], as well as at high prevalence in human sera [41]. However, in our study, members of *Parechovirus B* were absent in all screened rat or human samples. In comparison, we detected one sequence from rats (*Rattus argentiventer*) that may represent a new type of *Parechovirus C* [24].

Previously, there has been little study of picornavirus infection in bats. Picornavirus genomes (including those of kobuviruses, parechoviruses, cardioviruses, shanbaviruses and unclassified picornaviruses) were identified in bats in rural areas of mainland China and Hong Kong in two previous studies, although with a low prevalence and a low frequency of coinfection [30,42]. In our study, picornaviruses were found in 67% of bat samples, with 57% of these representing multiple picornavirus species detection from the same sample. All picornavirus sequences identified in bats as part of VISIONS were novel with respect to currently described species or types, suggesting that further work is needed to investigate picornavirus infection in bats and their role as a reservoir.

We observed a low prevalence of picornaviruses (2%) in the high-risk cohort. These were individuals presumed to be at high risk of zoonotic infections because of frequent and close occupational contact with animals, such as slaughterhouse workers and animal health workers. We also found that there was a clear distinction between human viruses and non-human animal viruses. No animal picornaviruses were detected in our human samples, including those picornaviruses known to infect humans via zoonotic transmission (including equine rhinitis A virus, equine rhinitis B virus, foot and mouth disease virus, and members of the species *Cardiovirus A*, *Cardiovirus B*, *Enterovirus E*, *Enterovirus H* and *Parechovirus B*) [7]. Similarly, although human enteroviruses can be detected in a range of animals, including bat, rat, pig, chicken and non-human primate [43,44,45,46] we did not detect any human picornaviruses [7] in animals, even when they had close human contacts. Therefore, our study suggests that the potential of picornavirus transmission between human and animals is low with members of different virus species restricted to a limited number of hosts.

In summary, our findings provide new information on the existence of novel viruses in a wide range of hosts. Our findings provide substantial new data on the prevalence, genetic diversity, and disease association of picornaviruses in several host populations at the animal–human interface. However, picornaviruses detected from animals were all taxonomically distinct from those found in humans, consistent with a relatively limited zoonotic potential of members of this virus family. In addition, we demonstrated the existence of several novel picornaviruses in rats and bats. Since these hosts can have close contract with humans, these novel animal viruses could be transmitted to humans, potentially becoming the origin of an emerging infectious disease.

## Data Availability

All picornavirus sequences determined in this study were deposited in GenBank under accession MZ544253-MZ544364. Metadata of picornavirus sequences found in this study and sequences from other study used in phylogenetic trees (Figure 5, Figure 6 and Figure 7) are listed in Supplementary Table S1.

## Supplementary Materials

The following are available online at https://www.mdpi.com/article/10.3390/v13091709/s1, Table S1: Sequences found in VIZIONS study and related sequences from Genbank.
