# Supplementary material for: No Exchange of Picornaviruses in Vietnam between Humans and Animals in a High-Risk Cohort with Close Contact despite High Prevalence and Diversity
Source: Viruses. 2021 Aug 27;13(9):1709. doi: 10.3390/v13091709 (PMC8473303; doi:10.3390/v13091709)
Supplement: Supplementary file 1 [file viruses-13-01709-s001.zip › viruses-1318070-supplementary.pdf]

Table S1 Sequences found in VIZIONS study (in black) and related sequences (in grey) from Genbank

Genus	cluster	virusname/Access.No	host.species	country	year
Hunnivirus	Hunnivirus|Rat-C1	16715x9-74	*Rattus argentiventer*	VietNam	2014
Hunnivirus	Hunnivirus|Rat-C1	16715x17-77	*Rattus argentiventer*	VietNam	2014
Hunnivirus	Hunnivirus|Rat-C1	KT944198.1	*Bandicota indica*	VietNam	2012
Hunnivirus	Hunnivirus|Rat-C1	22084x8-856	*Rattus argentiventer*	VietNam	2014
Hunnivirus	Hunnivirus|Rat-C1	KT944208.1	*Rattus argentiventer*	VietNam	2012
Hunnivirus	Hunnivirus|Rat-C1	22084x38-545	*Rattus argentiventer*	VietNam	2014
Hunnivirus	Hunnivirus|Rat-C1	KT944209.1	*Rattus argentiventer*	VietNam	2012
Hunnivirus	Hunnivirus|Rat-C1	22084x13-215	*Rattus argentiventer*	VietNam	2014
Hunnivirus	Hunnivirus|Rat-C1	16715x33-264	*Rattus argentiventer*	VietNam	2014
Hunnivirus	Hunnivirus|Rat-C1	KT944205.1	*Rattus tanezumi*	VietNam	2012
Hunnivirus	Hunnivirus|Rat-C1	16715x25-289	*Rattus argentiventer*	VietNam	2014
Hunnivirus	Hunnivirus|Rat-C1	16715x41-103	*Rattus argentiventer*	VietNam	2014
Hunnivirus	Hunnivirus|Rat-C1	16715x49-624	*Rattus argentiventer*	VietNam	2014
Hunnivirus	Hunnivirus|Rat-C1	16715x73-28	*Rattus argentiventer*	VietNam	2014
Hunnivirus	Hunnivirus|Rat-C1	22084x6-4204	*Rattus argentiventer*	VietNam	2014
Hunnivirus	Hunnivirus|Rat-C1	22084x4-164	*Rattus argentiventer*	VietNam	2014
Hunnivirus	Hunnivirus|Rat-C1	16715x81-177	*Rattus argentiventer*	VietNam	2014
Hunnivirus	Hunnivirus|Rat-C1	KT944202.1	*Rattus argentiventer*	VietNam	2012
Hunnivirus	Hunnivirus|Rat-C1	16715x89-10	*Rattus argentiventer*	VietNam	2014
Hunnivirus	Hunnivirus|Rat-C1	16715x25-288	*Rattus argentiventer*	VietNam	2014
Hunnivirus	Hunnivirus|Rat-C1	KT944203.1	*Rattus norvegicus*	VietNam	2012
Hunnivirus	Hunnivirus|Rat-C1	KT944207.1	*Rattus argentiventer*	VietNam	2012
Hunnivirus	Hunnivirus|Rat-C1	KT944214.1	*Rattus tanezumi*	VietNam	2013
Hunnivirus	Hunnivirus|Rat-C1	MF352430.1	*Rattus tanezumi*	China	2012
Hunnivirus	Hunnivirus|Rat-C2	16715x41-120	*Rattus argentiventer*	VietNam	2014
Hunnivirus	Hunnivirus|Rat-C2	16715x65-350	*Rattus argentiventer*	VietNam	2014
Hunnivirus	Hunnivirus|Rat-C2	KT944204.1	*Rattus argentiventer*	VietNam	2013
Hunnivirus	Hunnivirus|Rat-C2	22084x6-5619	*Rattus argentiventer*	VietNam	2014
Hunnivirus	Hunnivirus|Rat-C2	22084x22-123	*Rattus argentiventer*	VietNam	2014
Hunnivirus	Hunnivirus|Rat-C2	KT944212.1	*Rattus argentiventer*	VietNam	2012
Hunnivirus	Hunnivirus|Rat-C2	22057x58-10	*Rattus argentiventer*	VietNam	2014
Hunnivirus	Hunnivirus|Rat-C2	16715x33-233	*Rattus argentiventer*	VietNam	2014
Hunnivirus	Hunnivirus|Rat-C2	22056x78-1	*Rattus argentiventer*	VietNam	2014
Hunnivirus	Hunnivirus|Rat-C2	KT944213.1	*Rattus argentiventer*	VietNam	2012
Hunnivirus	Hunnivirus|Rat-C2	KT944206.1	*Rattus argentiventer*	VietNam	2012
Hunnivirus	Hunnivirus|Rat-C3	16715x57-54	*Rattus argentiventer*	VietNam	2014

Hunnivirus	Hunnivirus|Rat-C3	16715x89-9	*Rattus argentiventer*	VietNam	2014
Hunnivirus	Hunnivirus|Rat-C3	22084x7-3389	*Rattus argentiventer*	VietNam	2014
Hunnivirus	Hunnivirus|Rat-C3	22057x33-1	*Rattus argentiventer*	VietNam	2014
Hunnivirus	Hunnivirus|Rat-C3	22084x14-23	*Rattus argentiventer*	VietNam	2014
Hunnivirus	Hunnivirus|Rat-C3	KT944200.1	*Bandicota indica*	VietNam	2012
Hunnivirus	Hunnivirus|Rat-C3	KT944199.1	*Bandicota indica*	VietNam	2012
Hunnivirus	Hunnivirus|Rat-C4	KT944211.1	*Rattus norvegicus*	VietNam	2014
Hunnivirus	Hunnivirus|Rat-C4	KT944204.1	*Rattus norvegicus*	VietNam	2013
Hunnivirus	Hunnivirus|Rat-C4	KT944210.1	*Rattus norvegicus*	USA	2012
Hunnivirus	Hunnivirus|Rat-C4	MW417242.1	*Rattus norvegicus*	China	2017
Hunnivirus	Hunnivirus|Rat-C5	22084x6-2255	*Rattus argentiventer*	VietNam	2014
Hunnivirus	Hunnivirus|Rat-C5	22084x1-8350	*Rattus argentiventer*	VietNam	2014
Hunnivirus	Hunnivirus|Rat-C5	22054x5-16	*Rattus argentiventer*	VietNam	2013
Hunnivirus	Hunnivirus|Rat-C5	22084x1-20374	*Rattus argentiventer*	VietNam	2014
Hunnivirus	Hunnivirus|Rat-C5	22084x7-1893	*Rattus argentiventer*	VietNam	2014
Hunnivirus	HuV-A6	MF352413.1	*Niviventer confucianus*	China	2012
Hunnivirus	HuV-A6	KX156157.1	*Rodentia*	China	2015
Hunnivirus	Hunnivirus|Rat-C6	16715x18-31	*Rattus argentiventer*	VietNam	2014
Hunnivirus	Hunnivirus|Rat-C6	22084x8-313	*Rattus argentiventer*	VietNam	2014
Hunnivirus	Feline-hunnivirus	MF953886.2	*Felis catus*	China	2017
Hunnivirus	HuV-A1	NC_018668.1	*Bos taurus*	Hungary	2008
Hunnivirus	HuV-A2	HM153767.3	*Ovis aries*	Hungary	2009
Hunnivirus	HuV-A9	KY432925.1	*Rodentia*	China	2014
Parechovirus	Parechovirus-A1	17668x95-27	*Homo sapiens*	VietNam	2014
Parechovirus	Parechovirus-A1	19379x8-30	*Homo sapiens*	VietNam	2015
Parechovirus	Parechovirus-A1	16020x15-8	*Homo sapiens*	VietNam	2013
Parechovirus	Parechovirus-A1	16020x48-5	*Homo sapiens*	VietNam	2013
Parechovirus	Parechovirus-A1	16845x17-2	*Homo sapiens*	VietNam	2014
Parechovirus	Parechovirus-A1	19345x64-26	*Homo sapiens*	VietNam	2015
Parechovirus	Parechovirus-A1	17668x89-6	*Homo sapiens*	VietNam	2014
Parechovirus	Parechovirus-A1	19379x12-12	*Homo sapiens*	VietNam	2015
Parechovirus	Parechovirus-A1	19345x23-2	*Homo sapiens*	VietNam	2015
Parechovirus	Parechovirus-A1	16806x40-58	*Homo sapiens*	VietNam	2014
Parechovirus	Parechovirus-A1	16370x63-9	*Homo sapiens*	VietNam	2013
Parechovirus	Parechovirus-A1	19345x31-1	*Homo sapiens*	VietNam	2015
Parechovirus	Parechovirus-A1	16806x70-48	*Homo sapiens*	VietNam	2014
Parechovirus	Parechovirus-A1	19379x62-27	*Homo sapiens*	VietNam	2014
Parechovirus	Parechovirus-A1	16370x52-4	*Homo sapiens*	VietNam	2013

Parechovirus	Parechovirus-A1	L02971	*Homo sapiens*	USA	1992
Parechovirus	Parechovirus-A1	16318x54-27	*Homo sapiens*	VietNam	2014
Parechovirus	Parechovirus-A1	16370x64-9	*Homo sapiens*	VietNam	2013
Parechovirus	Parechovirus-A1	16806x9-1	*Homo sapiens*	VietNam	2013
Parechovirus	Parechovirus-A1	19344x62-6	*Homo sapiens*	VietNam	2014
Parechovirus	Parechovirus-A1	19379x60-27	*Homo sapiens*	VietNam	2015
Parechovirus	Parechovirus-A1	16806x76-33	*Homo sapiens*	VietNam	2014
Parechovirus	Parechovirus-A1	16317x75-2	*Homo sapiens*	VietNam	2014
Parechovirus	Parechovirus-A1	16806x82-189	*Homo sapiens*	VietNam	2013
Parechovirus	Parechovirus-A1	16845x1-6	*Homo sapiens*	VietNam	2014
Parechovirus	Parechovirus-A1	16806x27-1	*Homo sapiens*	VietNam	2014
Parechovirus	Parechovirus-C1	HF677705	*Apodemus sylvaticus*	Botambi	1972
Parechovirus	VZ-Rat	22057x67-9	*Rattus argentiventer*	VietNam	2014
Parechovirus	Parechovirus-E1	KY645497	*Falco vespertinus*	Hungary	2014
Parechovirus	Parechovirus-B1	AF327920	*Clethrionomys glareolus*	Sweden	1995
Parechovirus	Parechovirus|Bat-C1	16715x38-55	*Scotophilus kuhlii*	VietNam	2014
Parechovirus	Parechovirus|Bat-C1	17819x13-28	*Scotophilus kuhlii*	VietNam	2014
Parechovirus	Parechovirus|Bat-C1	16715x40-76	*Scotophilus kuhlii*	VietNam	2014
Parechovirus	Parechovirus|Bat-C1	16715x24-56	*Scotophilus kuhlii*	VietNam	2014
Parechovirus	Parechovirus|Bat-C1	17819x45-5	*Scotophilus kuhlii*	VietNam	2014
Parechovirus	Parechovirus|Bat-C1	16715x86-78	*Unknown bat*	VietNam	2014
Parechovirus	Parechovirus|Bat-C1	16715x78-129	*Scotophilus kuhlii*	VietNam	2014
Parechovirus	Parechovirus|Bat-C1	16715x14-80	*Scotophilus kuhlii*	VietNam	2014
Parechovirus	Parechovirus|Bat-C1	16845x63-120	*Scotophilus kuhlii*	VietNam	2014
Parechovirus	Parechovirus|Bat-C1	16715x78-130	*Scotophilus kuhlii*	VietNam	2014
Parechovirus	Parechovirus|Bat-C1	16845x31-17	*Scotophilus kuhlii*	VietNam	2014
Parechovirus	Parechovirus|Bat-C1	16715x39-32	*Scotophilus kuhlii*	VietNam	2014
Parechovirus	Parechovirus|Bat-C1	20724x87-644	*Scotophilus kuhlii*	VietNam	2015
Parechovirus	Parechovirus|Bat-C2	17819x11-22	*Scotophilus kuhlii*	VietNam	2014
Parechovirus	Parechovirus|Bat-C2	17819x16-21	*Scotophilus kuhlii*	VietNam	2014
Parechovirus	Parechovirus|Bat-C2	16715x13-17	*Unknown bat*	VietNam	2014
Parechovirus	Parechovirus|Bat-C2	16845x8-27	*Scotophilus kuhlii*	VietNam	2014
Parechovirus	Parechovirus|Bat-C2	20745x5-108	*Scotophilus kuhlii*	VietNam	2015
Parechovirus	Parechovirus|Bat-C3	17819x48-10	*Scotophilus kuhlii*	VietNam	2014
Parechovirus	Parechovirus|Bat-C3	16845x70-18	*Scotophilus kuhlii*	VietNam	2014
Parechovirus	Parechovirus|Bat-C3	20745x18-15	*Scotophilus kuhlii*	VietNam	2015
Parechovirus	Parechovirus|Bat-C3	20724x80-30	*Scotophilus kuhlii*	VietNam	2015
Parechovirus	Parechovirus|Bat-C3	17819x11-28	*Scotophilus kuhlii*	VietNam	2014

Parechovirus	Parechovirus|Bat-C3	20745x25-316	*Scotophilus kuhlii*	VietNam	2015
Parechovirus	Parechovirus|Bat-C3	16715x40-44	*Scotophilus kuhlii*	VietNam	2014
Parechovirus	Parechovirus|Bat-C3	16715x86-30	*Unknown bat*	VietNam	2014
Parechovirus	Parechovirus|Bat-C3	17819x20-34	*Scotophilus kuhlii*	VietNam	2014
Parechovirus	Parechovirus-D1	KF006989	*Mustela putorius furo*	Netherlands	2013
Parechovirus	Parechovirus-D	lMK348056.1	*Pipistrellus pipistrellus*	China	2016
Parechovirus	Parechovirus-F1	MG600084	*Teratoscincus roborowski*	China	2015
Cardiovirus	cardiovirus-D1	16020x71-3	*Homo sapiens*	VietNam	2013
Cardiovirus	cardiovirus-D1	EF165067	*homo sapiens*	USA	1981
Cardiovirus	cardiovirus-B1	JX443418	*Mus musculus*	USA	1952
Cardiovirus	cardiovirus-B2	M94868	*homo sapiens*	Russia	1955
Cardiovirus	cardiovirus-E1	KY432930	*Myodes andersoni*	China	2014
Cardiovirus	marmot-cardiovirus	KY855434	*Marmota himalayana*	China	2013
Cardiovirus	genet-fecal-theilovirus	KF823815	*Genetta genetta*	Spain	2012
Cardiovirus	cardiovirus-C1	JQ864242	*Rattus norvegicus*	USA	2010
Cardiovirus	cardiovirus-C2	JX683808	*Rattus norvegicus*	USA	2012
Cardiovirus	cardiovirus-C3	MF352424	*Rattus norvegicus*	China	2012
Cardiovirus	Cardiovirus|Rat-C3	16715x57-28	*Rattus argentiventer*	VietNam	2014
Cardiovirus	Cardiovirus|Rat-C3	16715x33-171	*Rattus argentiventer*	VietNam	2014
Cardiovirus	Cardiovirus|Rat-C2	22084x1-3992	*Rattus argentiventer*	VietNam	2014
Cardiovirus	Cardiovirus|Rat-C1	22084x5-1820	*Rattus argentiventer*	VietNam	2014
Cardiovirus	Cardiovirus|Rat-C1	22084x8-300	*Rattus argentiventer*	VietNam	2014
Cardiovirus	Cardiovirus|Rat-C1	22084x1-3987	*Rattus argentiventer*	VietNam	2014
Cardiovirus	Cardiovirus|Rat-C1	KT944132.1	*Bandicota indica*	VietNam	2012
Cardiovirus	Cardiovirus|Rat-C1	KT944133.1	*Rattus argentiventer*	VietNam	2012
Cardiovirus	Cardiovirus|Rat-C1	16715x33-215	*Rattus argentiventer*	VietNam	2014
Cardiovirus	cardiovirus-A1	M81861	*Rattus norvegicus*	NA	1992
Cardiovirus	cardiovirus-A2	JX257003	*Apodemus sylvaticus*	Germany	2005
Cardiovirus	cardiovirus-F1	KY432929	*Myodes andersoni*	China	2014
Mupivirus	Mupivirus|Rat-C1	22057x86-7	*Rattus argentiventer*	VietNam	2014
Mupivirus	Mupivirus|Rat-C1	16715x73-23	*Rattus argentiventer*	VietNam	2014
Mupivirus	Mupivirus|Rat-C1	16715x10-93	*Rattus argentiventer*	VietNam	2014
Mupivirus	Mupivirus|Rat-C1	22056x85-3	*Rattus losea*	VietNam	2014
Mupivirus	Mupivirus|Rat-C1	22084x13-82	*Rattus argentiventer*	VietNam	2014
Mupivirus	mupivirus-A2	KY432934.1	*Niventer confucianus*	China	2015
Mupivirus	mupivirus-A1	KY432924.1	*Mus caroli*	China	2014
Mosavirus	Mosavirus|Rat-C1	22084x10-70	*Rattus argentiventer*	VietNam	2014

Mosavirus	Mosavirus|Rat-C1	22084x1-1109	*Rattus argentiventer*	VietNam	2014
Mosavirus	Mosavirus|Rat-C1	22084x6-985	*Rattus argentiventer*	VietNam	2014
Mosavirus	Mosavirus|Rat-C1	22084x7-1073	*Rattus argentiventer*	VietNam	2014
Mosavirus	MoV-A1	JF973687.1	*Peromyscus crinitus*	USA	2010
Mosavirus	MoV-A2	KF958461.1	*Coracias garrulus*	Hungary	2011
Mosavirus	MoV-B1	KY855435.1	*Marmota himalayana*	China	2013
Unclassified	Unclassified|Bat-C1	16715x40-55	*Scotophilus_kuhlii*	VietNam	2014
Unclassified	Unclassified|Bat-C1	16845x14-8	*Scotophilus_kuhlii*	VietNam	2014
Unclassified	Unclassified|Bat-C1	20745x7-311	*Scotophilus_kuhlii*	VietNam	2015
Unclassified	Unclassified|Bat-C1	16845x77-12	*Scotophilus_kuhlii*	VietNam	2014
Unclassified	Unclassified|Bat-C1	16845x22-8	*Scotophilus_kuhlii*	VietNam	2014
Unclassified	Unclassified|Bat-C1	16845x56-22	*Scotophilus_kuhlii*	VietNam	2014
Unclassified	Unclassified|Bat-C1	20745x13-25	*Scotophilus_kuhlii*	VietNam	2015
Unclassified	Unclassified|Bat-C1	16715x14-68	*Scotophilus_kuhlii*	VietNam	2014
Unclassified	Unclassified|Bat-C1	16845x64-33	*Scotophilus_kuhlii*	VietNam	2014
Unclassified	Unclassified|Bat-C1	16715x40-52	*Scotophilus_kuhlii*	VietNam	2014
Unclassified	Unclassified|Bat-C1	16715x54-52	*Scotophilus_kuhlii*	VietNam	2014
Unclassified	Unclassified|Bat-C1	16715x45-12	*Scotophilus_kuhlii*	VietNam	2014
Unclassified	Unclassified|Bat-C1	16715x38-16	*Scotophilus_kuhlii*	VietNam	2014
Unclassified	Unclassified|Bat-C1	16715x55-49	*Unknown bat*	VietNam	2014
Unclassified	Unclassified|Bat-C1	16715x71-53	*Scotophilus_kuhlii*	VietNam	2014
Unclassified	Unclassified|Bat-C1	16845x40-27	*Scotophilus_kuhlii*	VietNam	2014
Unclassified	Ia-io-picornavirus-1	JQ814852.1	*Unknown bat*	China	2010
